# Acute kidney injury and its predictors among HIV-positive patients in Africa: Systematic review and meta-analysis

**DOI:** 10.1371/journal.pone.0298302

**Published:** 2024-02-09

**Authors:** Abere Woretaw Azagew, Hailemichael Kindie Abate, Yohannes Mulu Ferede, Chilot Kassa Mekonnen

**Affiliations:** Department of Medical Nursing, School of Nursing, College of Medicine and Health Sciences, University of Gondar, Gondar, Ethiopia; University of the Witwatersrand, SOUTH AFRICA

## Abstract

**Background:**

cute kidney injury(AKI) is a rapid loss of the kidney’s excretory function, resulting in an accumulation of end products of nitrogen metabolism. The causes of AKI in HIV-positive patients are not well investigated, but it may be associated with antiretroviral drug side effects and HIV itself. Even though there were studies that reported the prevalence of AKI among HIV-positive patients in Africa, their findings were inconsistent across the studies.

**Methods:**

We searched on PubMed, Embas, Ebsco, OVID, Cochrane Library, and other supplementary search engines, including Google and Google Scholar. Articles published upto July 2023 were included in this review study. The quality of the study was assessed using the Newcastle-Ottawa Scale for cross-sectional, case-control, and cohort studies. The data were extracted using a Microsoft Excel spreadsheet and exported to Stata version 14 for analysis. A random effect meta-analysis model was used to estimate the pooled prevalence of AKI among HIV-positive patients. Heterogeneity was evaluated using Cochrane Q statistics and I squared (I^2^). Furthermore, the graphic asymmetric test of the funnel plot and/or Egger’s tests were computed to detect publication bias. Sensitivity analysis was computed to see the effect of a single study on the summary effects. To treat the publication bias, a trim and fill analysis was carried out. The protocol of this review has been registered in an international database, the Prospective Register of Systematic Reviews (PROSPERO),with reference number CRD42023446078.

**Results:**

A total of twenty-four original articles comprising 7913HIV-positive patients were included in the study. The pooled prevalence of AKI among HI-positive patients was found to be 23.35% (95% CI: 18.14–28.56%, I^2^ = 97.7%, p-value <0.001). Low hemoglobin (Hgb <8mg/dl) was found to be the determinant factor for AKI among HIV-positive patients (AOR = 2.4; 95% CI:1.69–3.4, I^2^ = 0.0%, p-value = 0.40). In meta-regression analysis, sample size was the possible source of variation among the included studies (AOR = 3.11, 95%CI: 2.399–3.83).

**Conclusions:**

The pooled prevalence of AKI among HIV-positive patients was high. HIV-positive patients with low hemoglobin levels are at risk of developing AKI. Hence, regular monitoring of kidney function tests is needed to prevent or delay the risk of AKI among HIV-positive patients. Healthcare workers should provide an integrated healthcare service to HIV-positive patients on the prevention, treatment, and reduction of the progression of AKI to advanced stages and complications.

## Background

The human immunodeficiency virus (HIV) is still the leading challenge worldwide [1, 2]. Globally, 39 million people were living with HIV at the end of 2022. Africa remains the most severely affected region, with nearly one in every twenty-five adults living with HIV and accounting for more than two-thirds of the people living with HIV worldwide [3].

Renal dysfunction, especially acute kidney injury (AKI), is an important cause of hospitalization and mortality among HIV-positive patients. It is a common complication in HIV-positive patients [4]. Injury or diseases, including HIV infection, can damage the kidneys and lead to kidney diseases. In people with HIV, poorly controlled HIV infection and co-infection increase the risk of AKI [5].

The exact causes of HIV-associated AKI are not well investigated, but it is associated with volume depletion, sepsis, and the intake of nephrotoxic medications [6]. It is also associated with individual risk factors, HIV-correlated factors, and antiretroviral drug toxicity [7]. AKI can lead to life-threatening complications such as end-stage renal failure, volume overload, electrolyte disturbance, and multi-organ dysfunction [8].

AKI can affect the entire population, but its severity is higher among HIV-positive patients. The death rate of AKI among HIV-positive patients was 21.2%, 25%, and 35.3%, compared with HIV-negative individualsat10%, 23.3%, and 16% at one, two, and five-year follow-up periods [9]. HIV-positive patients with advanced disease, advanced age, pre-existing kidney diseases, and concomitant use of nephrotoxic medication are at increased risk of adverse renal events [10].

In Africa, even though there were different studies associated with acute kidney injury among HIV-positive patients, the findings were inconsistent across the studies. Therefore, this study aimed to estimate the prevalence and identify predictors of AKI among HIV-positive patients in Africa.

## Methods

### Study protocol registration and reporting

The protocol has been registered on PROSPERO with reference number CRD42023446078. The reporting of this review follows the Preferred Reporting Item for Systematic Review and Meta-Analysis (PRISMA-2020) checklist [11] (S1 File).

### Study design and search strategies

We searched on PubMed, Embas, Ebsco, OVID, Cochrane Library, and other supplementary search engines: Google and Google Scholar. Endnote Version 7 reference management software was used to download, organize, review, de-duplicate, and cite the articles. Our comprehensive search strategies were carried out using controlled vocabularies such as medical subject headings(MeSH) terms. Boolean logic operators “AND” and “OR” were used to combine search terms. The search string is stated as “Acute kidney injury” OR AKI OR “renal impairment” OR “renal dysfunction” OR “renal disease” AND “Human Immuno-deficiency Virus patients” OR“HIV patients” OR “HIV positive patient*”OR“Sero-positive patients” OR“Acquired Immune Deficiency Syndrome patients” OR“AIDS patients” OR “people living with HIV/AIDS” OR PLWHA AND Africa (S2 File). Articles were searched by title (ti), abstract (ab), and full text (ft). Modifications of the search results were made by limiters such as study design and country. Articles published up to July 2023 were included in the study. Two reviewers (HMK and CKM) independently searched and screened articles by title, abstract, and full text. The disagreements between the reviewers were resolved through discussion. Further disagreements were solved by the involvement of the third person.

### Inclusion and exclusion criteria

The eligibility criteria of the included studies are summarized in the table below(Table 1).

**Table 1:** Inclusion and exclusion criteria for included research articles.

Criteria	Inclusion criteria	Exclusion criteria
Population	HIV positive patients	Chronic kidney diseases prior to the exposure of HIV
	People living with HIV/AIDS	
		Patients with dialysis and/or renal dysfunction before the onset of HIV
Age	Adult (age ≥18 years)	
Design	Observational study designs (cross-sectional, cohort, case-control, and survey)	
Publication status	Both published and/or unpublished studies such as preprints	Qualitative studies
		Conference papers
		Articles with no full text
Country	African countries	Scoping review
		Narrative review
		Systematic review and meta-analysis
Publication year	Articles published up-to July 2023	
Language	Any language	

Notes: AIDS-Acquired Immune-Deficiency Syndrome, HIV-Human Immuno-deficiency Virus

### Outcome measurement

Acute kidney injury is defined as an increase in serum creatinine of 3 mg/dl within 48 hours and/or 1.5 times the baseline within the previous seven days, as well as urine output of < 0.5 mg/kg/hour for six hours [12].

### Data extraction

The data was extracted using a Microsoft Excel spreadsheet. The format was prepared by all the authors and piloted for its clarity, aim, consistency, and depth to capture information from the included articles. Using the data extraction format, information such as author(s), year of publication, study design, country of the study, method of data collection, funding status, sample size, prevalence or incidence of AKI, and effect estimates such as OR with a 95% CI were extracted (S3 File). Two reviewers (HMK and CKM)independently screened the articles by titles, abstracts, and full texts. Possible inconveniencies were solved by discussion with the reviewers and/or the involvement of the third person.

### Quality appraisal

Articles were assessed for their quality using the Newcastle-Ottawa assessment scale adapted from cross-sectional [13], cohort [14], and case-control studies [15]. A score of 6 or above was considered a high-quality article. Two reviewers (AWA and YMF) assessed the quality of the articles. The reviewers compared the quality of the appraisal scores and resolved inconsistencies before calculating the final appraisal score.

### Data analysis

The extracted data was exported to Stata version 14 for analysis. Heterogeneity was detected by Cochrane Q statistics and I^2^. The heterogeneity test statistics results of below 25%, 50%, and above 75% were declared as low, moderate, and high heterogeneity [16], respectively. A random effect meta-analysis model was used to estimate the pooled prevalence of AKI [17]. The graphic asymmetry of the funnel plot test and/or Egger’s test (p-value <0.005) were used to detect the publication bias [18].

## Results

### Study selection and characteristics of included studies

**Fig 1:**
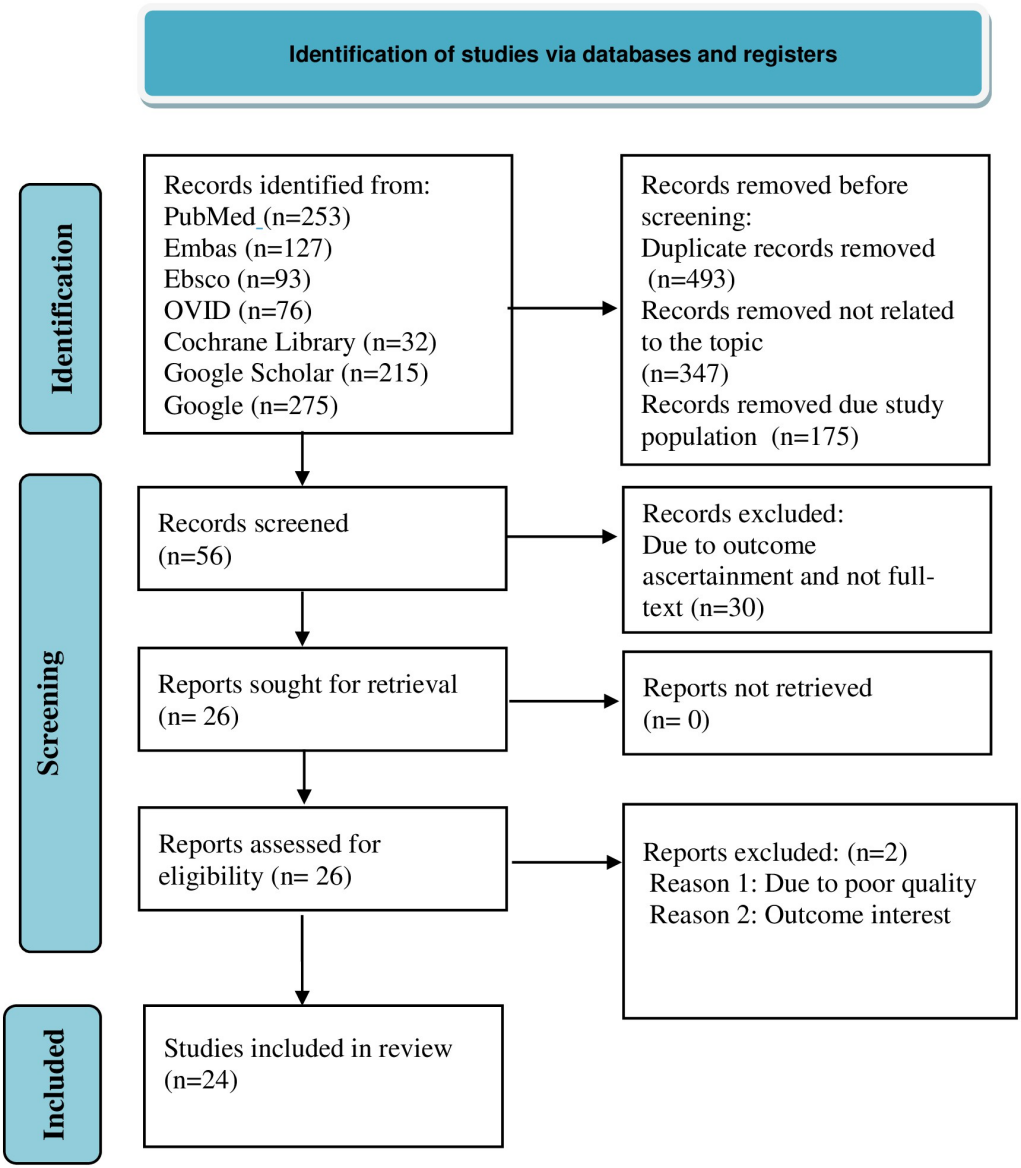
PRISMA flow chart for flow of information through the phases of systematic review.

The search strategy retrieved 1071 original articles. About 493, 347, and 175 articles were removed due to duplication, not related to the topic of interest, and population variation, respectively. About 56 articles remained, and following further screening, 30 articles were removed due to variations in outcome ascertainment and not full-text. Then 26 full-text articles were assessed for eligibility, of which two articles were excluded because of poor quality and outcome interest (Fig 1). Finally, twenty-four articles were retrieved and included in the review, with a total of 9713 populations. Of the twenty-four studies, five were from Ethiopia [19–23], four from Tanzania [24–27], four from Nigeria [28–31], two from Uganda [32, 33], one from Kenya [34], one from Cameroon [35], one from South Africa [36], one from Malawi [37], one from Senegal [38], one from Zambia [39], one from Rwanda [40], one from Burkina Faso [41], and one from DR Congo [42]. The pooled prevalence was calculated from the aforementioned studies, whereas for predictors of AKI, three studies for hemoglobin level [19, 35, 41], six studies for CD4 count [19, 20, 24–26, 31], and three studies for WHO clinical HIV staging [20, 26, 35] were used. The prevalence of AKI among HIV patients ranged from 2.53%in Uganda [33] to 56.8% in Nigeria [31]. The majority of the studies used lab tests as a method of data collection. All of the included studies had high-quality scores(Table 2).

**Table 2:** Characteristics and quality status of included studies.

Authors/year	Country	population	Study design	Definition Basis	Data collection	Funding	Sample size	Prevalence (%)	Quality score
Alex MT et al. 2022 [35]	Cameroon	HIV	Cohort	KDIGO -2012	Lab test, interview	Not reported	206	30.6	7
Fiseha T &Gebreweld A. 2021 [19]	Ethiopia	HIV	Cohort	eGFR	Record review	Not funded	353	22.1	8
Karoney MJ et al.2022 [34]	Kenya	HIV	Cross-sectional	eGFR	Lab test, interview	EDCTP	261	10	9
Kefeni BT, et al. (2021) [20]	Ethiopia	HIV	Cross-sectional	eGFR	Record review, lab test	JU	352	20.7	7.5
Kimweri D, et al. (2021) [32]	Uganda	HIV	Cohort	KDIGO	Interview, record review, lab test	not funded	384	19.2	8
Mwanja MN et al. (2022) [24]	Tanzania	HIV	Cross-sectional	eGFR	Record review, lab test	TFEL	396	20.7	8.5
Mwemezi O, et al. (2020) [25]	Tanzania	HIV	Cross-sectional	EGFR	Record review, lab test	Not funded	287	32.8	9
Mugabo C &Ndikubwimana I. (2023) [40]	Rwanda	HIV	Cohort	eGFR	record review	Not reported	98	24.4	8.5
Fall K et al. (2017) [38]	Senegal	HIV	Cohort	eGFR	Interview, record review, lab test	not reported	248	12.9	8
Nyende L et al. (2020) [33]	Uganda	HIV	Cross- sectional	eGFR	Record review	Mulago KCCA project	278	2.53	7
Enyew K, et al. (2016) [21]	Ethiopia	HIV	Cross-sectional	eGFR	lab test	AAU	60	23.3	7.5
Mapesi H, et al. (2021) [26]	Tanzania	HIV	Cohort	eGFR	lab test	MOH T	556	7.4	7
Vachiat AI, et al. (2013) [36]	South Africa	HIV	cross-sectional	eGFR	Record review	Not reported	101	21	8
Ali Y, et al. (2012) [23]	Ethiopia	HIV	Cross-sectional	eGFR	Interview, record review, lab test	Not reported	321	15.9	9
Semde A, et al. (2021) [41]	Burkina Faso	HIV	Cross-sectional	KDIGO	Record review	Not reported	364	29.94	8.5
Emem CP, et al. (2008) [28]	Nigeria	HIV	Cross- sectional	eGFR	lab test	Not reported	400	38	7
Maina SM et al. (2023) [30]	Nigeria	HIV	Case-control	Renal Biopsy	lab test	Not reported	200	10.5	7.5
Struik GM, et al. (2015) [37]	Malawi	HIV	Cross-sectional	Cockcroft–Gault equation	lab test	Not reported	526	23.3	8
Yilma D, et al. (2012) [22]	Ethiopia	HIV	Cross-sectional	eGFR	lab test	FAD	340	26.9	9
Okafor UH, et al. (2015) [29]	Nigeria	HIV	Cross-sectional	eGFR	Interview, record review, lab test	Not reported	383	53.3	9
Sakajiki MA, et al. (2011) [31]	Nigeria	HIV	Cross-sectional	eGFR	Interview, record review, lab test	Not reported	240	56.8	7.5
Kilonzo BS, et al. (2016) [27]	Tanzania	HIV	Cross-sectional	eGFR	Interview, record review, lab test	Not reported	637	28	8
Tembo S, et al. (2017) [39]	Zambia	HIV	Cross-sectional	eGFR	lab test	Not reported	360	24.76	9
Ekat MH, et al. (2012) [42]	DR Congo	HIV	Cross-sectional	eGFR	lab test	No reported	562	8.5	8.5

**Notes**:- EDC: European and Developing Countries, eGFR: estimated Glomerular Filtration Rate, KDIGO: Kidney disease Improving Global Outcome, HIV: Human immunodeficiency virus; TFEL-Tanzania Field Epidemiology and Laboratory, MOH T- Ministry of Health Tanzania, FAD-Foreign affairs of Denmark

### The prevalence of acute kidney injury

**Fig 2:**
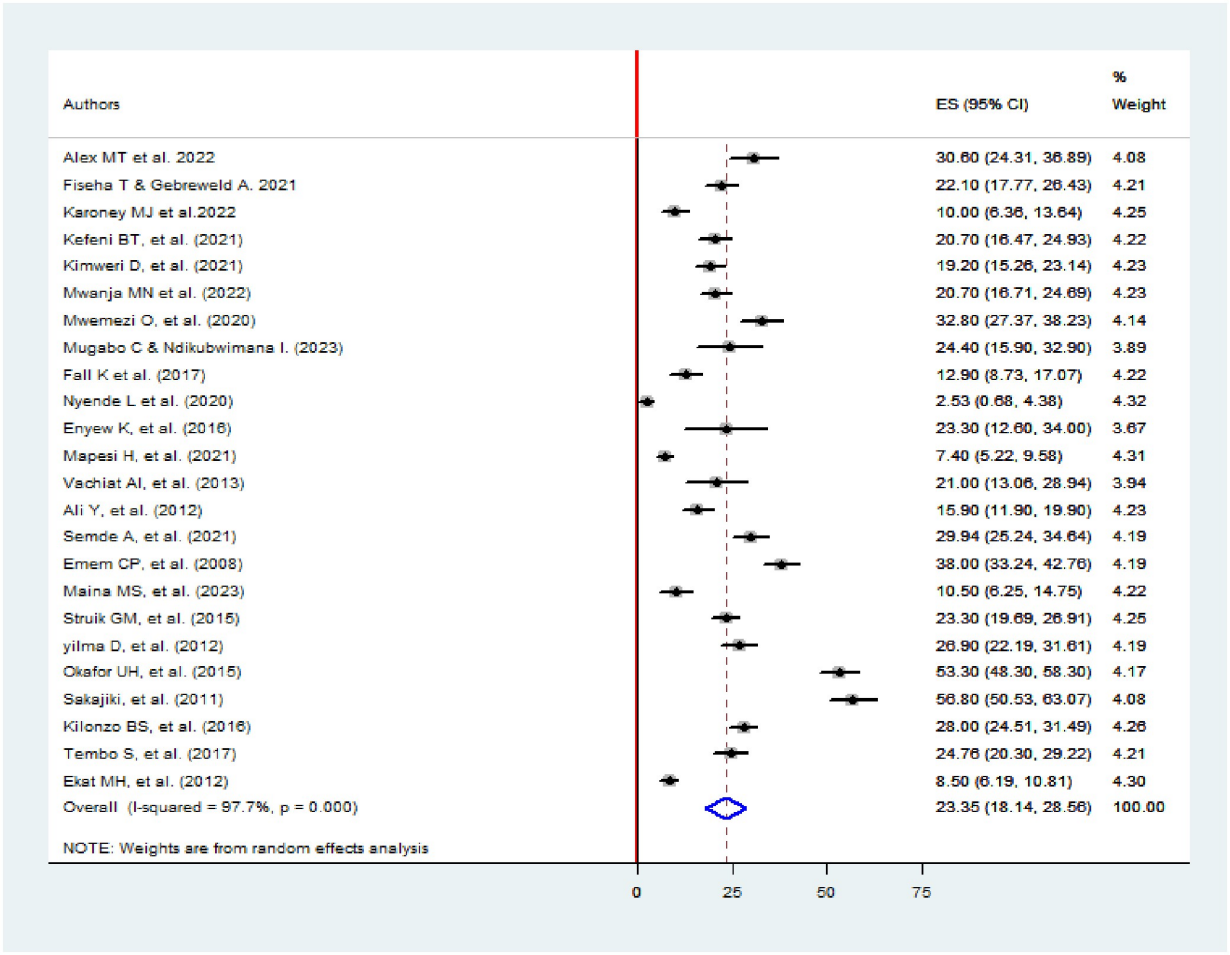
The forest plot shows the pooled prevalence of AKI among HIV patients in Africa.

The pooled prevalence of AKI among HIV patients was found to be 23.35% (95% CI: 18.14–28.56%, I^2^ = 97.7%, p-value <0.001) (Fig 2).

### Subgroup analysis

**Fig 3:**
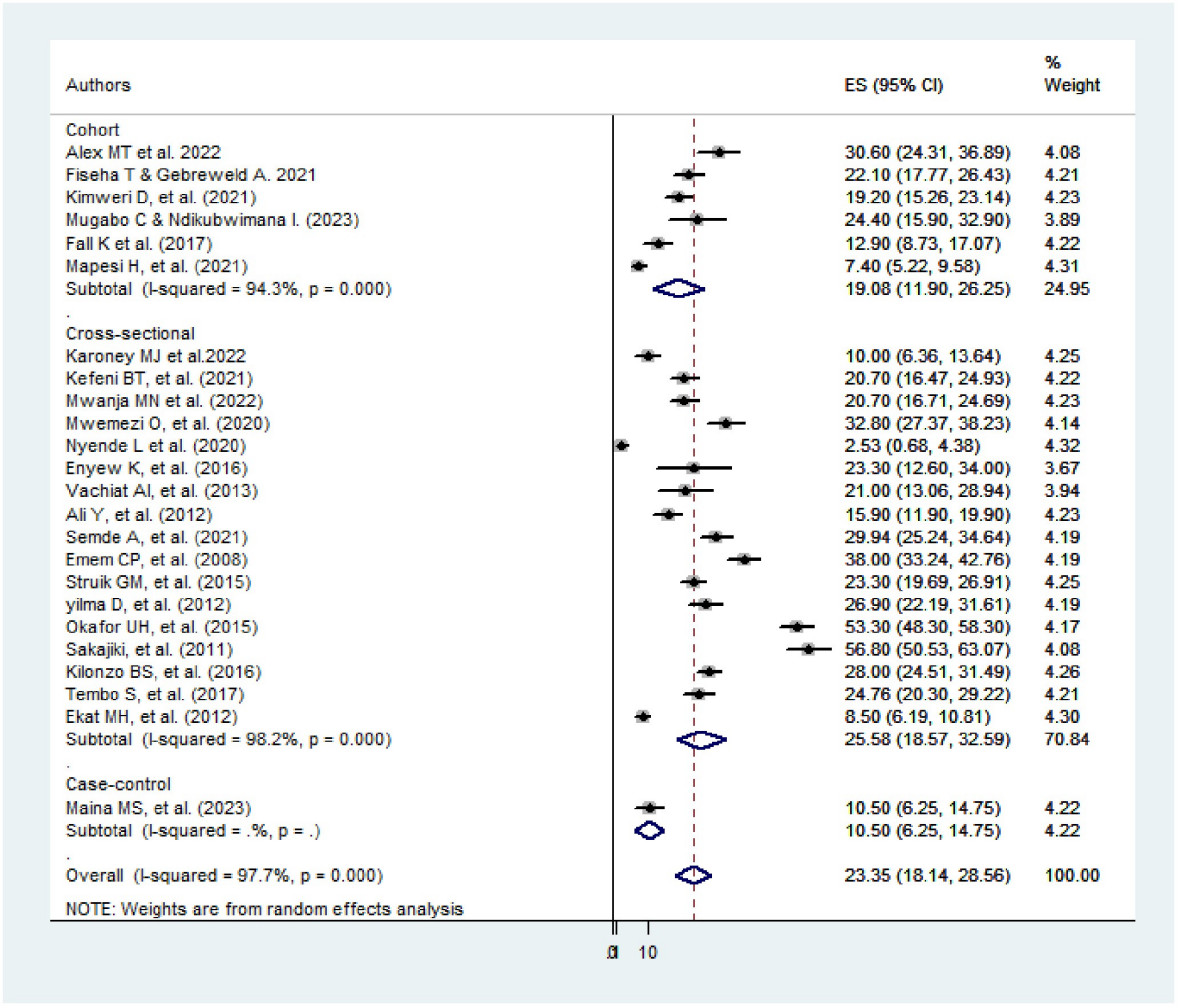
Subgroup analysis by study design among the included studies.

The subgroup analysis was computed using the study designs of the included studies. In the subgroup analysis, the level of heterogeneity is high. In the cohort designs, the level of heterogeneity was 94.3%, whereas in cross-sectional designs it was 98.2% (Fig 3). Therefore, meta-regression analyses need to be computed to identify the real source of the variations.

### Meta-regression analysis

To identify the causes of the covariates, both bivariate and multivariate meta-regression analyses were computed. In the multivariate analyses, sample size was found to be the cause of the variations (AOR = 3.11, 95%CI: 2.399–3.83) (Table 3).

**Table 3:** Meta-regression analysis for variation of the included studies.

Variables	Std.err	p-value	Coef.	95%CI
Publication year	0.584	0.174	0.82	0.39–1.203
Sample size	0.345	0.001	3.11	2.399–3.83

**Notes**: CI-confidence interval, Coef.-coefficient, Std.err- Standard error

### Publication bias

**Fig 4:**
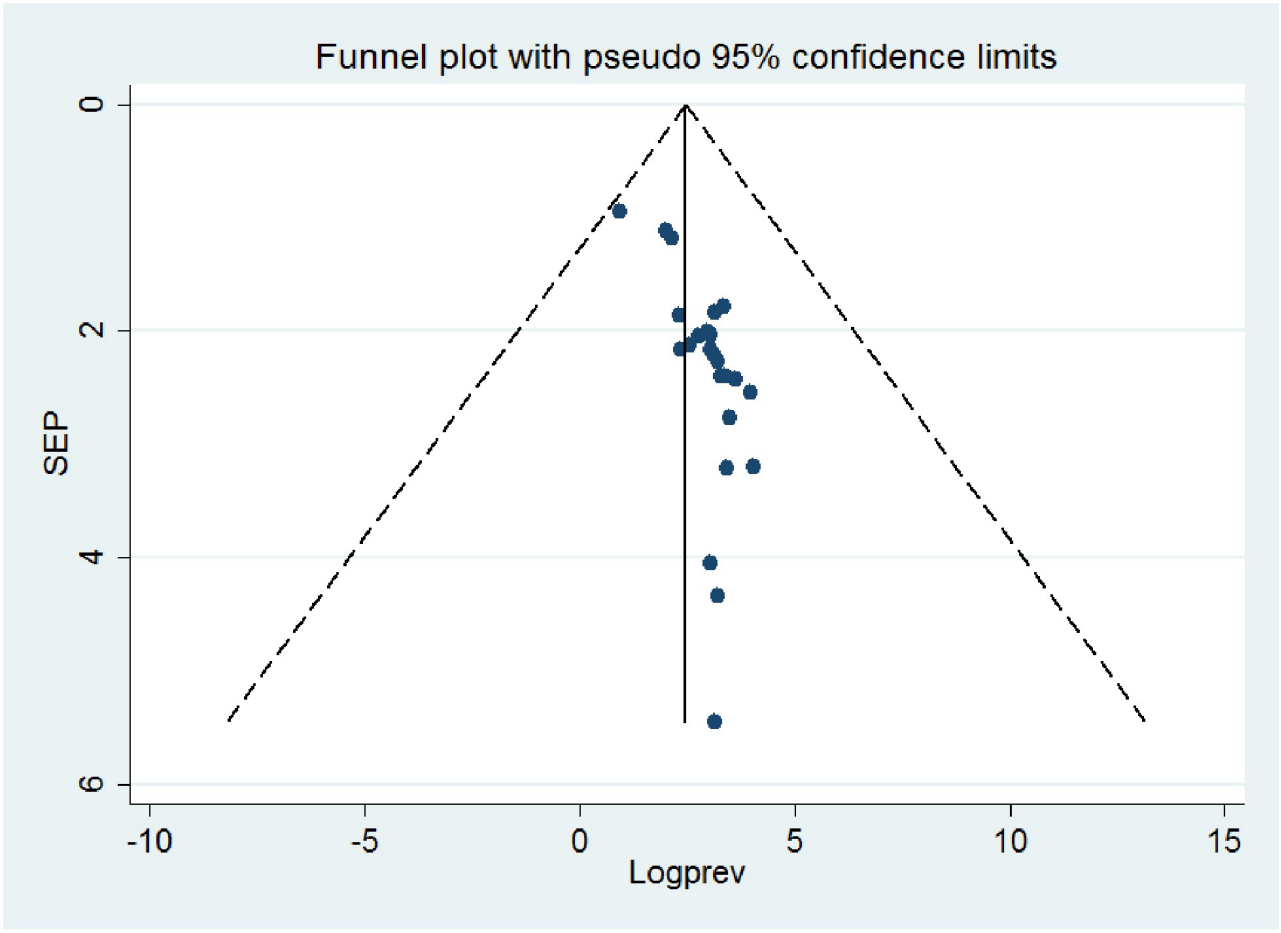
Funnel plot to assess the publication bias of the included studies.

**Fig 5:**
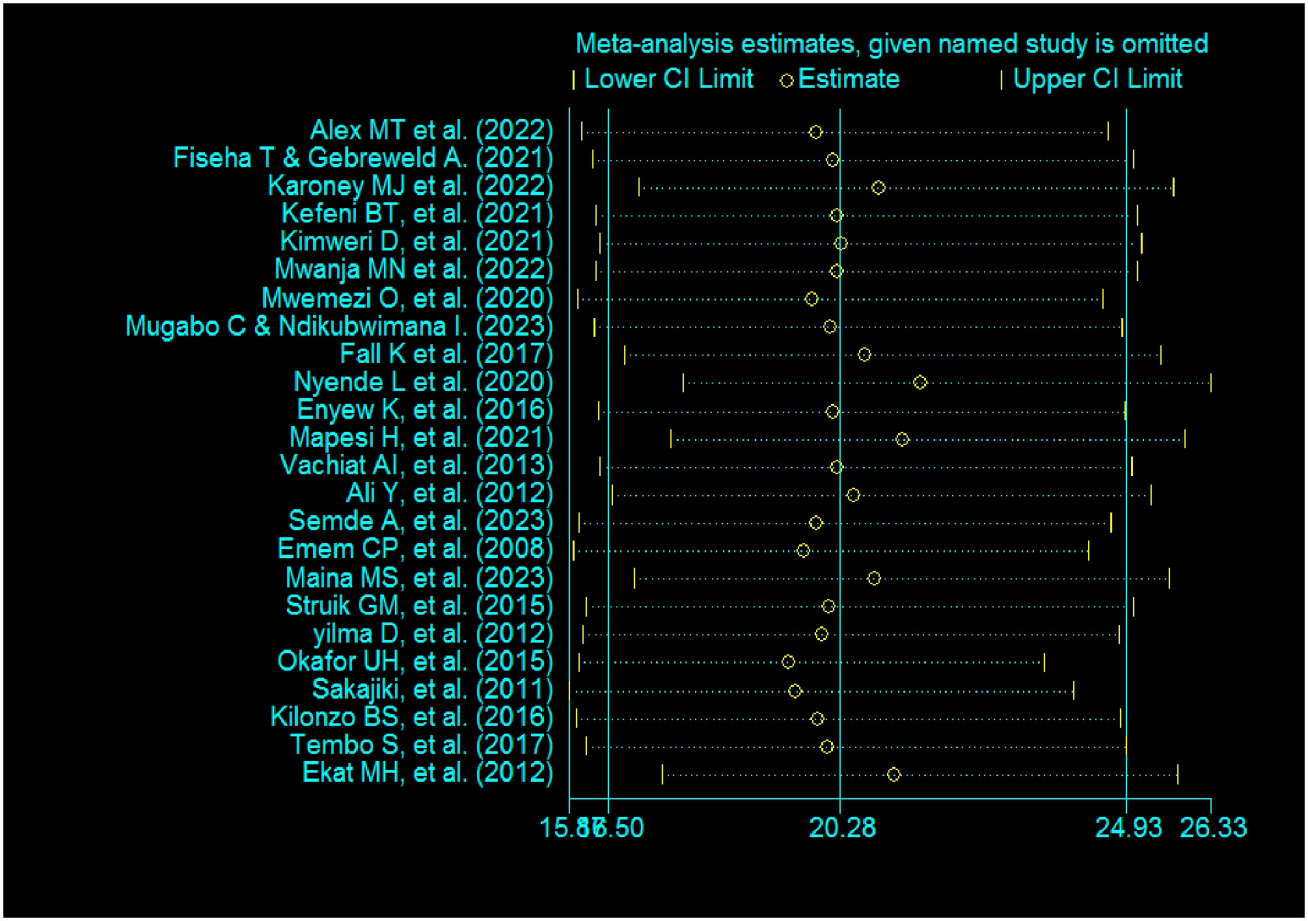
Sensitivity analysis among the included study to detect the effect of single study.

**Fig 6:**
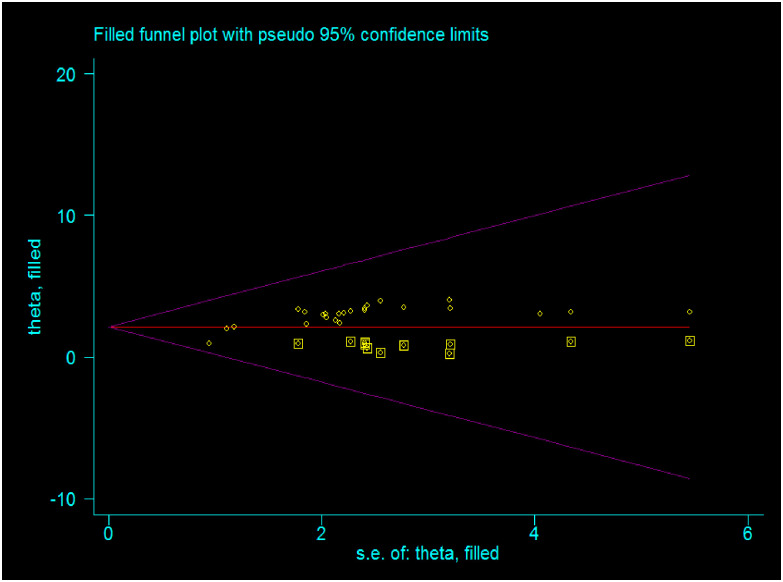
Trim and fill analysis results among articles.

The publication bias was assessed by the graphic asymmetry test of the funnel plot and/or Egger’s tests. The funnel plot test showed that there was an asymmetrical distribution, indicating there is a publication bias (Fig 4). Similarly, Egger’s test shows that the p-value is <0.001, indicating there is publication bias. Sensitivity analysis was computed to see the effect of a single study on the summary effect estimates, indicating there is no single study effect or outlier (Fig 5). To treat small study effects, non-parametric trim and fill analyses were computed. As a result, eleven articles were filled, making a total of 35 articles with 34 degrees of freedom, a p-value of 1.00, and a moment-based estimate of between-study variance of 0.00(Fig 6).

### Predictors of AKI among HIV patients

#### Hemoglobin level

**Fig 7:**
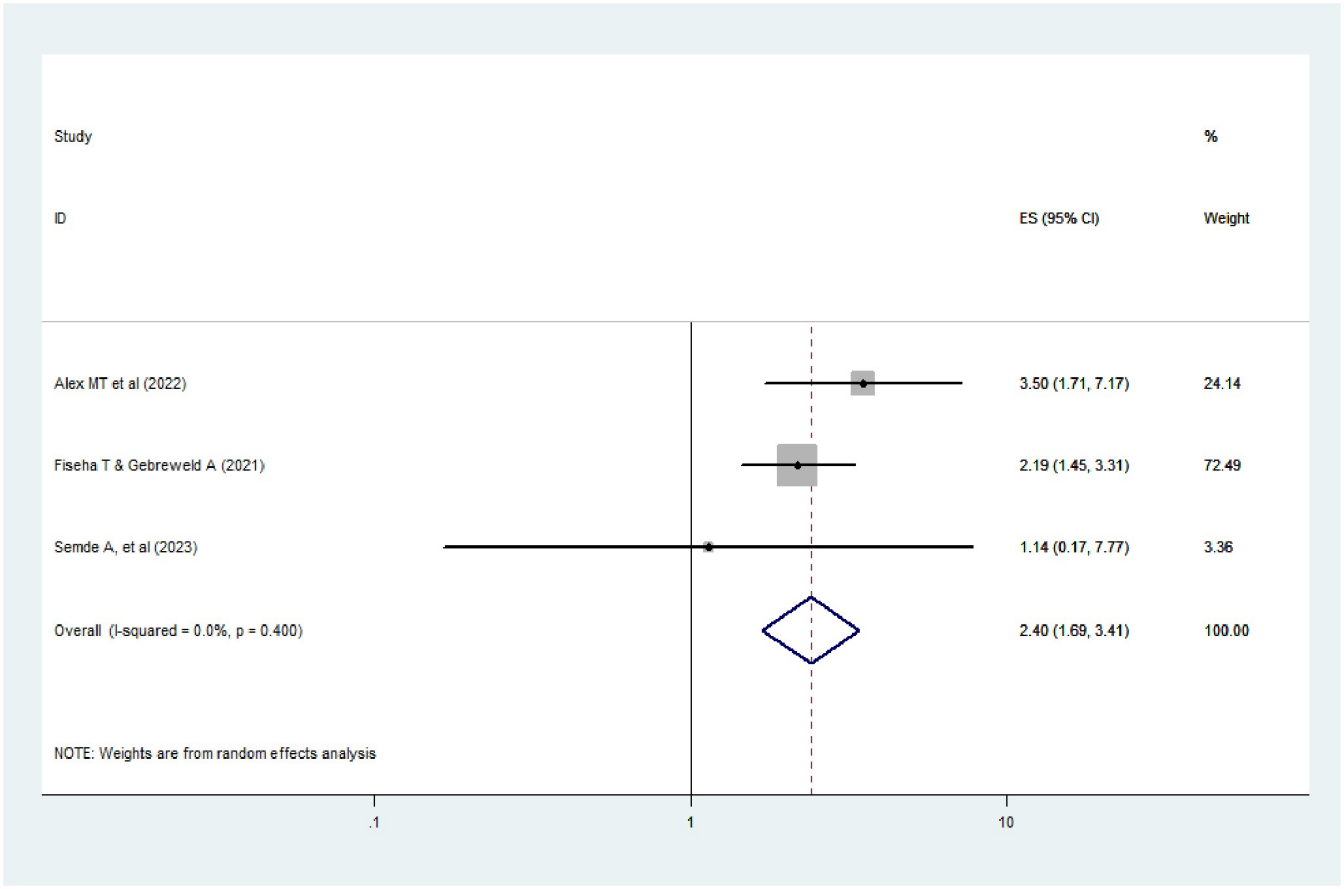
The effect of hemoglobin on AKI among HIV positive patients.

In this review study, having low hemoglobin (hgb<8mg/dl) was found to be the determinant factor of AKI among HIV-positive patients (AOR = 2.4; 95% CI:1.69–3.4, I^2^ = 0.0%, p-value = 0.40).From the forest plot, the I^2^ was 0.0% and the p-value was 0.40 (Fig 7), indicating there is no variation across the studies.

#### CD4 count

**Fig 8:**
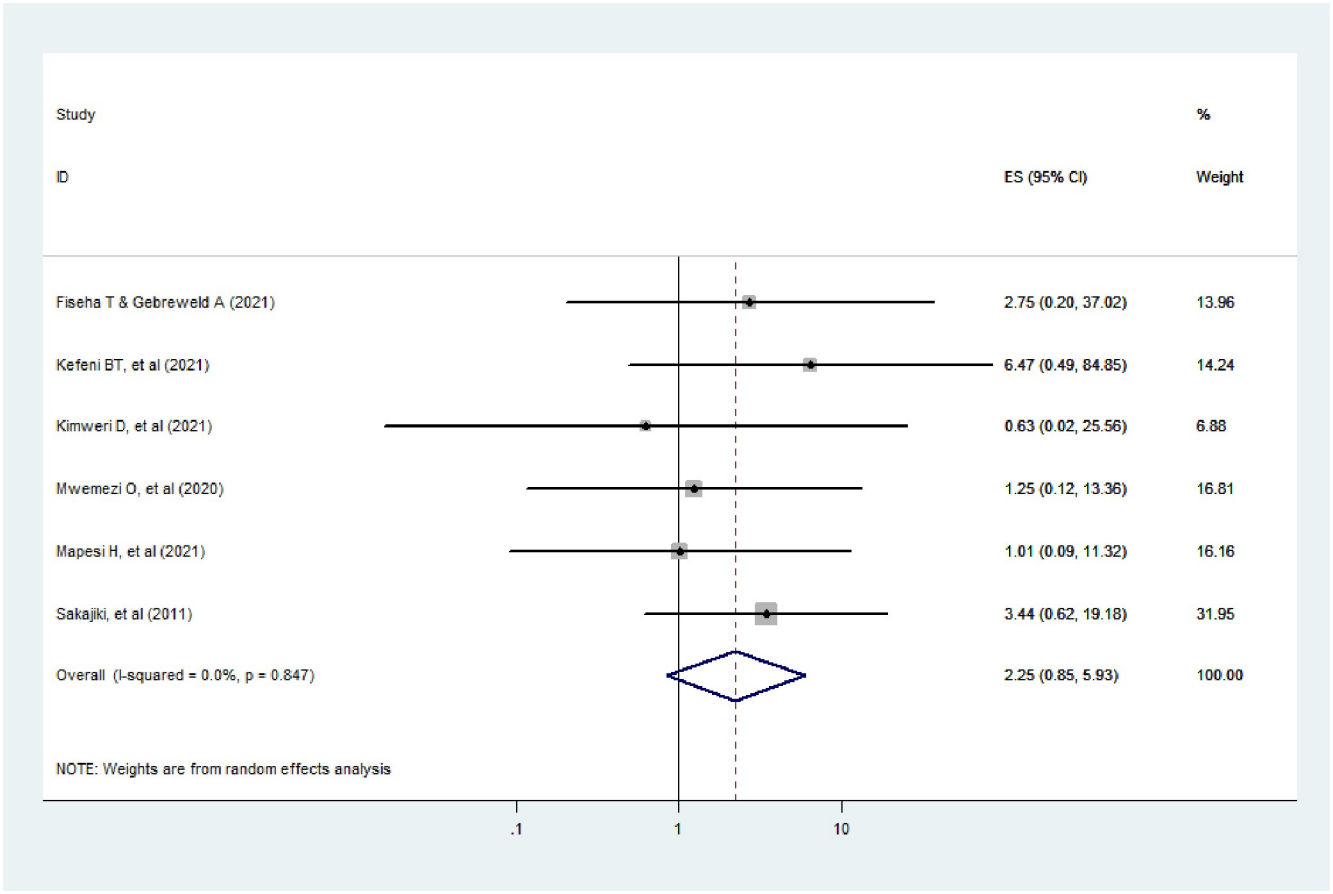
The effect of CD4 count on AKI among HIV positive patients.

The findings of this study revealed that a low CD4 count is not a risk factor for AKI among HIV patients(AOR = 2.25; 95% CI: 0.85–5.93, I^2^ = 0.0%, p-value = 0.847). As shown in the figure below, the I^2^ was 0.0% and the p-valuewas0.847, meaning that there was no variation across the included studies (Fig 8).

#### WHO clinical HIV staging

**Fig 9:**
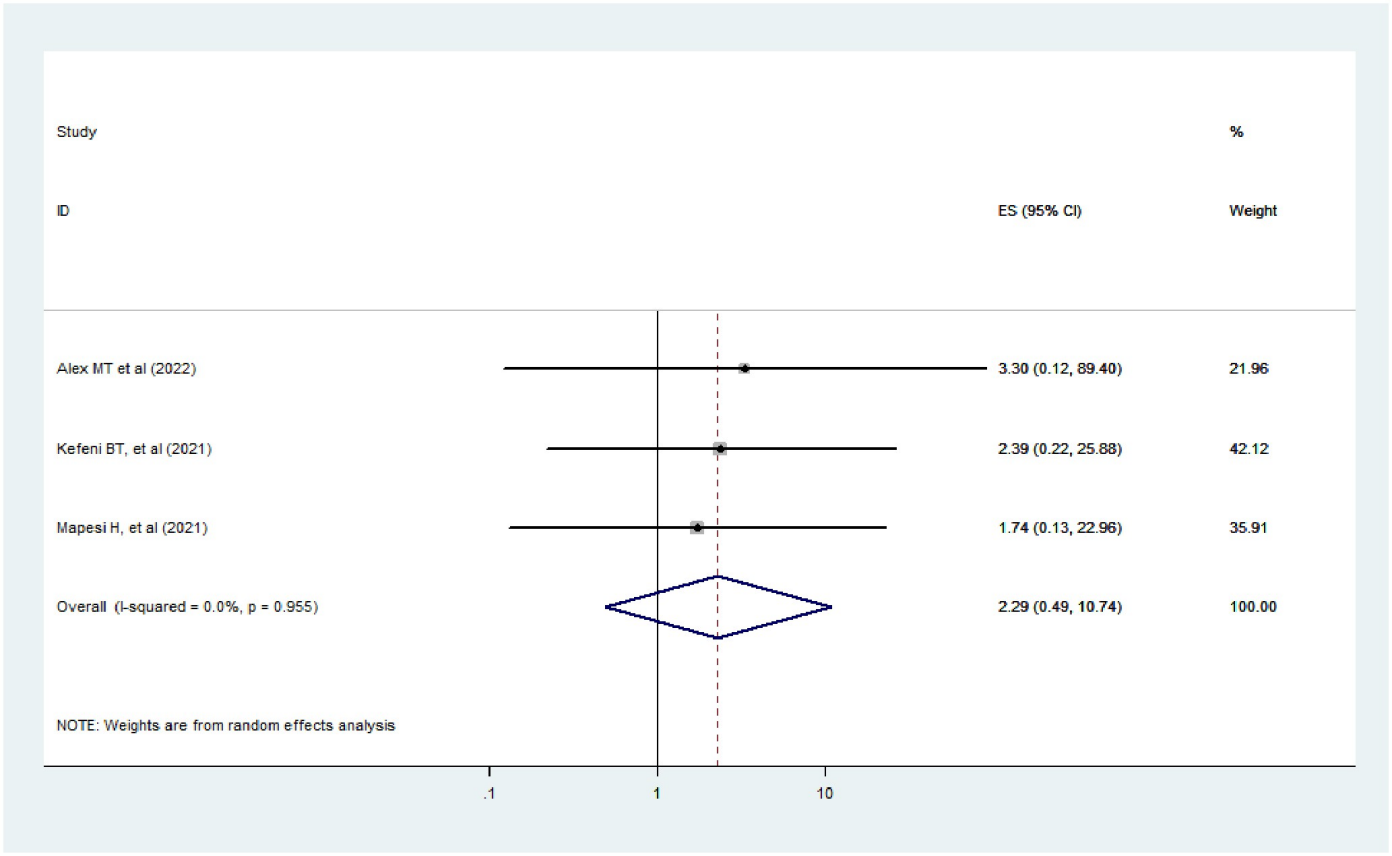
The effect of WHO clinical staging on AKI among HIV positive patients.

The study depicts that the WHO clinical HIV stage is not a predictor for AKI among HIV-positive patients(AOR = 2.29, 95% CI: 0.49–10.74, I^2^ = 0.0%, p-value = 0.955) (Fig 9). From the forest plot, the I^2^ was 0.00% and the p-value was 0.955, which indicates there is no variation across the study.

## Discussion

The findings of this review study revealed that the pooled prevalence of AKI among HIV-positive patients in Africa was 23.35% (95% CI: 18.14–28.56%). The result of this study is lower than the studies conducted in Shanghai, China 12.5% [43] and 0.7%Edmonton, Canada [44]. The possible reason for the discrepancy is that the Chinese study focused on incidence and used a variety of outcome measurement criteria, whereas the Canadian study focused on the effect of Tenofovir Disoproxil Fumarate (TDF) on the development of AKI and used clinical trial studies, whereas the current study used observational studies.

A low hemoglobin level was found to be the determinant factor of AKI among HIV-positive patients. HIV-positive patients who had a low hemoglobin level (hgb<8mg/dl) were2.4 times more likely to develop AKI compared to normal hemoglobin levels. This is supported by a study in Seoul [45], which stated that the odds of AKI are associated with decreased hemoglobin. The possible reasons that renal insufficiency is associated with anemia (low hemoglobin) [46]. A low hemoglobin level decreases renal tissue oxygenation and results in acute tubular necrosis.

The sample size was one of the possible sources of variation across the studies. In the meta-regression analysis, sample size is the source of the variation for the included studies. This is due to the fact that when the sample size is too small, the bias from sampling will increase the frequency of sampling [47]. Larger sample sizes are required to attain the desired normal power [48]. Additionally, the small sample size resulted ina small effect size, which can lead to the possibility of variation.

As a strength, the authors investigate HIV-associated AKI in Africa at large. In addition, the study investigates both the prevalence and associated factors of AKI among HIV-positive patients.

The study has the following limitations: Firstly, the study did not assess the stage of AKI, mortality, or its treatment outcome. Therefore, we recommendedthat further global researches which need to be carried out to estimate the global prevalence and identify other important predictors of HIV-associated AKI among HIV-positive populations.

### Implications of the study

The findings of this study provide input for healthcare workers to provide an integrated HIV care service along with routine ART services. The study gives direction for healthcare workers to emphasize on regular monitoring of renal function tests to HIV-positive patients. Additionally, the study also helps policy makers and decision-makers focus on intervention strategies for the early detection and prevention of AKI in HIV positives.

## Conclusions

This systematic review and meta-analysis revealed that the pooled prevalence of AKI among HIV-positive patients in Africa was high. HIV-positive patients with low hemoglobin levels are at risk for developing AKI. Hence, regular monitoring of kidney function tests is needed to prevent, detect, and treat AKI. Therefore, healthcare workers should provide integrated HIV-renal healthcare services such as renal function tests to prevent, detect, and treat AKIto reduce its progression to advanced stages and complications.

## Supporting information

S1 File PRISMA checklist. (DOCX)

S2 File Search strategy. (DOCX)

S3 File Data availability statement. (DOCX)

## Abbreviations

AKI: Acute Kidney Injury
AOR: Adjusted Odds Ratio
CI: Confidence Interval
HIV: Human immunodeficiency virus
PRISMA: Preferred Reporting Item for Systematic Review and Meta-Analysis
WHO: World Health Organization

## References

[1] Demberg T, Robert-Guroff M. Controlling the HIV/AIDS epidemic: current status and global challenges. Frontiers in immunology. 2012;3:250. doi: 10.3389/fimmu.2012.00250 22912636 PMC3418522

[2] Tian X, Chen J, Wang X, Xie Y, Zhang X, Han D, et al. Global, regional, and national HIV/AIDS disease burden levels and trends in 1990–2019: A systematic analysis for the global burden of disease 2019 study. Frontiers in Public Health. 2023;11:1068664. doi: 10.3389/fpubh.2023.1068664 36875364 PMC9975742

[3] Global HIV & AIDS statistics—Fact sheet [Internet]. 2022.

[4] Jones R, Scott C, Nelson M, Levy J. Renal complications in HIV. International journal of clinical practice. 2007;61(6):991–8. doi: 10.1111/j.1742-1241.2007.01376.x 17504361

[5] Wyatt CM. Kidney disease and HIV infection. Topics in antiviral medicine. 2017;25(1):13. 28402929 PMC5677039

[6] Kalim S, Szczech LA, Wyatt CM, editors. Acute kidney injury in HIV-infected patients. Seminars in nephrology; 2008: Elsevier. 10.1016/j.semnephrol.2008.08.008 PMC2676161 19013326

[7] Maggi P, Bartolozzi D, Bonfanti P, Calza L, Cherubini C, Di Biagio A, et al. Renal complications in HIV disease: between present and future. AIDS reviews. 2012;14(1):37–53. 22297503

[8] Levey AS, James MT. Acute kidney injury. Annals of internal medicine. 2017;167(9):ITC66–ITC80. doi: 10.7326/AITC201711070 29114754

[9] Lopes JA, Melo MJ, Raimundo M, Fragoso A, Antunes F. Long-term risk of mortality for acute kidney injury in HIV-infected patients: a cohort analysis. BMC nephrology. 2013;14:1–7. 23394360 10.1186/1471-2369-14-32 PMC3574852

[10] Boswell M, Rossouw T. Approach to acute kidney injury in HIV-infected patients in South Africa. South Afr J HIV Med. 2017; 18 (1): 714. doi: 10.4102/sajhivmed.v18i1.714 29568636 PMC5843257

[11] Page MJ, McKenzie JE, Bossuyt PM, Boutron I, Hoffmann TC, Mulrow CD, et al. The PRISMA 2020 statement: an updated guideline for reporting systematic reviews. International journal of surgery. 2021;88:105906. doi: 10.1016/j.ijsu.2021.105906 33789826

[12] Makris K, Spanou L. Acute kidney injury: definition, pathophysiology and clinical phenotypes. The clinical biochemist reviews. 2016;37(2):85. 28303073 PMC5198510

[13] Moskalewicz A, Oremus M. No clear choice between Newcastle–Ottawa Scale and Appraisal Tool for Cross-Sectional Studies to assess methodological quality in cross-sectional studies of health-related quality of life and breast cancer. Journal of clinical epidemiology. 2020;120:94–103. 31866469 10.1016/j.jclinepi.2019.12.013

[14] Wells G, Shea B, O’Connell D, Peterson J, Welch V, Losos M, et al. Newcastle-Ottawa quality assessment scale cohort studies. University of Ottawa. 2014.

[15] Peterson J, Welch V, Losos M, Tugwell P. The Newcastle-Ottawa scale (NOS) for assessing the quality of nonrandomised studies in meta-analyses. Ottawa: Ottawa Hospital Research Institute. 2011;2(1):1–12.

[16] Rücker G, Schwarzer G, Carpenter JR, Schumacher M. Undue reliance on I 2 in assessing heterogeneity may mislead. BMC medical research methodology. 2008;8(1):1–9. 19036172 10.1186/1471-2288-8-79 PMC2648991

[17] Borenstein M, Hedges LV, Higgins JP, Rothstein HR. A basic introduction to fixed‐effect and random‐effects models for meta‐analysis. Research synthesis methods. 2010;1(2):97–111. doi: 10.1002/jrsm.12 26061376

[18] Begg CB, Mazumdar M. Operating characteristics of a rank correlation test for publication bias. Biometrics. 1994:1088–101. 7786990

[19] Fiseha T, Gebreweld A. Renal function in a cohort of HIV-infected patients initiating antiretroviral therapy in an outpatient setting in Ethiopia. PloS one. 2021;16(1):e0245500. doi: 10.1371/journal.pone.0245500 33481839 PMC7822244

[20] Kefeni BT, Hajito KW, Getnet M. Renal Function Impairment and Associated Factors Among Adult HIV-Positive Patients Attending Antiretroviral Therapy Clinic in Mettu Karl Referral Hospital: Cross-Sectional Study. HIV/AIDS-Research and Palliative Care. 2021:631–40. doi: 10.2147/HIV.S301748 34135641 PMC8200135

[21] Eneyew K, Seifu D, Amogne W, Menon M. Assessment of renal function among HIV-Infected patients on combination antiretroviral therapy at Tikur Anbessa Specialized Hospital, Addis Ababa, Ethiopia. Technology and Investment. 2016;7(3):107–22.

[22] Yilma D, Abdissa A, Kæstel P, Tesfaye M, Olsen MF, Girma T, et al. Renal function in Ethiopian HIV-positive adults on antiretroviral treatment with and without tenofovir. BMC infectious diseases. 2020;20(1):1–11. doi: 10.1186/s12879-020-05308-9 32762646 PMC7409649

[23] Yishak Ali DY, Fasl Tessema,. The prevalence and predictors of renal dysfunction in Human Immuno-deficiency virus positive people at Jimma University specialized Hospital South west Ethiopia. 2012.

[24] Mwanjala MN, Urio LJ, Mtebe MV. Prevalence and predictors of renal dysfunction among people living with HIV on antiretroviral therapy in the Southern Highland of Tanzania: a hospital-based cross-sectional study. Pan African Medical Journal. 2022;41(1). doi: 10.11604/pamj.2022.41.137.27025 35519167 PMC9034560

[25] Mwemezi O, Ruggajo P, Mngumi J, Furia FF. Renal dysfunction among HIV-infected patients on antiretroviral therapy in Dar es Salaam, Tanzania: a cross-sectional study. International journal of nephrology. 2020;2020. 10.1155/2020/8378947 PMC7568141 33101732

[26] Mapesi H, Okuma J, Franzeck F, Wilson HI, Senkoro E, Byakuzana T, et al. Prevalence, incidence and predictors of renal impairment in persons with HIV receiving protease-inhibitors in rural Tanzania. Plos one. 2021;16(12):e0261367. doi: 10.1371/journal.pone.0261367 34910776 PMC8673654

[27] Kilonzo SB, Seiffudin AT, Bakshi FA, Gunda DW. Renal dysfunction among adult patients in Mwanza, Tanzania: prevalence, outcomes and associated factors. Tanzania Journal of Health Research. 2016;18(3).

[28] Emem CP, Arogundade F, Sanusi A, Adelusola K, Wokoma F, Akinsola A. Renal disease in HIV-seropositive patients in Nigeria: an assessment of prevalence, clinical features and risk factors. Nephrology Dialysis Transplantation. 2008;23(2):741–6. doi: 10.1093/ndt/gfm836 18065807

[29] Okafor U, Unuigbe E, Chukwuonye E. Prevalence, clinical and laboratory characteristics of kidney disease in antiretroviral naïve HIV infected patients in South-South Nigeria. African Journal of Nephrology. 2015;18(1):12–7. 10.4103/1319-2442.174155 26787579

[30] Sulaiman MM, Shettima J, Ummate I, Arogundade FA, Yusuph H, Nwankwo E, et al. Comparative Evaluation of Prevalence, Risk Factors, and Pathologic Features of Kidney Disease in Highly Active Antiretroviral Therapy-Naive and Highly Active Antiretroviral Therapy-Experienced Patients at a Tertiary Health Facility in Maiduguri, Northeastern Nigeria. Saudi Journal of Kidney Diseases and Transplantation. 2022;33(1):72–9. doi: 10.4103/1319-2442.367828 36647981

[31] Mohammad AS. Prevalence Risk factors and patterns of Kidney disease in patients with HIV/AIDS at Amino Kano Teaching hospital: A clinico-pathologic study. Fuculty of Internal Medicine. 2011.

[32] Kimweri D, Ategeka J, Ceasor F, Muyindike W, Nuwagira E, Muhindo R. Incidence and risk predictors of acute kidney injury among HIV-positive patients presenting with sepsis in a low resource setting. BMC nephrology. 2021;22:1–5. 34187389 10.1186/s12882-021-02451-6 PMC8243728

[33] Nyende L, Kalyesubula R, Sekasanvu E, Byakika-Kibwika P. Prevalence of renal dysfunction among HIV infected patients receiving Tenofovir at Mulago: a cross-sectional study. BMC nephrology. 2020;21(1):1–6. doi: 10.1186/s12882-020-01873-y 32571236 PMC7310064

[34] Karoney MJ, Koech MK, Njiru EW, Owino Ong’or WD. Proximal tubular renal dysfunction among HIV infected patients on Tenofovir versus Tenofovir sparing regimen in western Kenya. Plos one. 2022;17(9):e0273183. doi: 10.1371/journal.pone.0273183 36108078 PMC9477312

[35] Alex MT, Carelle TNA, Maimouna M, Georges TD, Enow AG. Incidence, risk factors, and outcomes of acute kidney injury among HIV positive medical admissions at the Bamenda Regional Hospital. Journal of Clinical Nephrology. 2022;6(2):068–73.

[36] Vachiat A, Musenge E, Wadee S, Naicker S. Renal failure in HIV-positive patients–A South African experience. Clin Kidney J. 2013; 6 (6): 584–589. doi: 10.1093/ckj/sft128 26069826 PMC4438376

[37] Struik G, Den Exter R, Munthali C, Chipeta D, Van Oosterhout J, Nouwen J, et al. The prevalence of renal impairment among adults with early HIV disease in Blantyre, Malawi. International journal of STD & AIDS. 2011;22(8):457–62. doi: 10.1258/ijsa.2011.010521 21795419

[38] Fall K, Cissé MM, Lemrabott AT, Faye M, Dial MC, Faye A, et al. Renal Disease among HIV Positive Patients in Senegal. Open Journal of Nephrology. 2017;7(4):101–6.

[39] Tembo S, Chirwa L, Musonda P, Mulenga L, Mweemba A. The Prevalence of Kidney Dysfunction and Associated Risk Factors among ART-Naïve HIV-1 infected Adults at the University Teaching Hospital in Lusaka, Zambia. Medical Journal of Zambia. 2017;44(3):176–83.

[40] Ndikubwimana MCa. Assessment of the Effects of Antiretroviral Medications on Kidney Status in Rwanda. Advances in Clinical Toxicology. 2023(2577–4328).

[41] Aoua S, Idrissa K, Judicaël D, Gaoussou S. Epidemiology, Clinical Presentation and Outcome of Acute Kidney Injury in the City of Bobo-Dioulasso (Burkina Faso). Health Sciences and Disease. 2023;24(1).

[42] Ekat M.H. C C, Diafouka M., Akolbout M., Mahambou-Nsonde D., Nzounza P., Simon B., et al. Prevalence and factors associated with renal disease among patients with newly diagnoses of HIV in Brazzaville, Republic of Congo. Medecine et Sante Tropicales 2012;2013(23):176–80. 10.1684/mst.2013.0170 23787222

[43] Shi R, Chen X, Lin H, Ding Y, He N. Incidence of impaired kidney function among people with HIV: a systematic review and meta-analysis. BMC nephrology. 2022;23(1):107. doi: 10.1186/s12882-022-02721-x 35300612 PMC8932163

[44] Cooper RD, Wiebe N, Smith N, Keiser P, Naicker S, Tonelli M. Systematic review and meta-analysis: renal safety of tenofovir disoproxil fumarate in HIV-infected patients. Clinical Infectious Diseases. 2010;51(5):496–505. doi: 10.1086/655681 20673002

[45] Han SS, Baek SH, Ahn SY, Chin HJ, Na KY, Chae D-W, et al. Anemia is a risk factor for acute kidney injury and long-term mortality in critically ill patients. The Tohoku journal of experimental medicine. 2015;237(4):287–95. doi: 10.1620/tjem.237.287 26607258

[46] Fiseha T, Tamir Z, Seid A, Demsiss W. Prevalence of anemia in renal insufficiency among HIV infected patients initiating ART at a hospital in Northeast Ethiopia. BMC Hematol 2017; 17: 1. doi: 10.1186/s12878-017-0071-2 28116101 PMC5240406

[47] Li S-j, Jiang H, Yang H, Chen W, Peng J, Sun M-w, et al. The dilemma of heterogeneity tests in meta-analysis: a challenge from a simulation study. PLoS One. 2015;10(5):e0127538. doi: 10.1371/journal.pone.0127538 26023932 PMC4449216

[48] Vallejo G, Ato M, Fernández MP, Livacic-Rojas PE. Sample size estimation for heterogeneous growth curve models with attrition. Behavior Research Methods. 2019;51:1216–43. doi: 10.3758/s13428-018-1059-y 29934696

