# Supplementary material for: Acute kidney injury and its predictors among HIV-positive patients in Africa: Systematic review and meta-analysis
Source: PLoS One. 2024 Feb 9;19(2):e0298302. doi: 10.1371/journal.pone.0298302 (PMC10857608; doi:10.1371/journal.pone.0298302)
Supplement: S1 File — (DOCX) [file pone.0298302.s001.docx]

| **Section and Topic** | **Item #** | **Checklist item** | **Location where item is reported** |
| --- | --- | --- | --- |
| **TITLE** | | |  |
| Title | 1 | **Acute kidney injury and its predictors among HIV-positive patients in Africa: Systematic review and Meta-analysis** | 1 |
| **ABSTRACT** | | |  |
| Abstract | 2 | **Background:** Acute kidney injury (AKI) is a rapid loss of the kidney’s excretory function, resulting in an accumulation of end products of nitrogen metabolism. The causes of AKI in HIV-positive patients are not well investigated, but it may be associated with antiretroviral drug side effects and HIV itself. Even though there were studies that reported the prevalence of AKI among HIV-positive patients in Africa, their findings were inconsistent across the studies.  **Methods**: We searched on PubMed, Embas, Ebsco, OVID, Cochrane Library, and other supplementary search engines, including Google and Google Scholar. Articles published up to July 2023 were included in this review study. The quality of the study was assessed using the Newcastle-Ottawa Scale for cross-sectional, case-control, and cohort studies. The data were extracted using a Microsoft Excel spreadsheet and exported to Stata version 14 for analysis. A random effect meta-analysis model was used to estimate the pooled prevalence of AKI among HIV-positive patients. Heterogeneity was evaluated using Cochrane Q statistics and I squared (I^2^). Furthermore, the graphic asymmetric test of the funnel plot and/or Egger’s tests were computed to detect publication bias. Sensitivity analysis was computed to see the effect of a single study on the summary effects. To treat the publication bias, a trim and fill analysis was carried out. The protocol of this review has been registered in an international database, the Prospective Register of Systematic Reviews (PROSPERO), with reference number CRD42023446078.  **Results**: A total of twenty-four original articles comprising 7913 HIV-positive patients were included in the study. The pooled prevalence of AKI among HI-positive patients was found to be 23.35% (95% CI: 18.14-28.56%, I^2^ = 97.7%, p-value <0.001). Low hemoglobin (Hgb <8 mg/dl) was found to be the determinant factor for AKI among HIV-positive patients (AOR = 2.4; 95% CI: 1.69-3.4, I^2^ = 0.0%, p-value = 0.40). In meta-regression analysis, sample size was the possible source of variation among the included studies (AOR = 3.11, 95% CI: 2.399-3.83).  **Conclusions**: The pooled prevalence of AKI among HIV-positive patients was high. HIV-positive patients with low hemoglobin levels are at risk of developing AKI. Hence, regular monitoring of kidney function tests is needed to prevent or delay the risk of AKI among HIV-positive patients. Healthcare workers should provide an integrated healthcare service to HIV-positive patients on the prevention, treatment, and reduction of the progression of AKI to advanced stages and complications. | 2 |
| **INTRODUCTION** | | |  |
| Rationale | 3 | Kidney diseases are one of the most serious public health issues in the world, with increased morbidity, mortality, and health-care costs. Acute kidney injury is a rapid loss of the kidney’s excretory function resulting in an accumulation of end product of nitrogen metabolism such as urea and creatinine and/or a decrease urine output within three months. The cause of acute kidney injury is multi-factoral and is associated with pre-renal, intrinsic/intra-renal, and post-renal causes. In Ethiopia, there are inconsistent findings regarding the prevalence of acute kidney injury. | 3 |
| Objectives | 4 | To estimate the pooled prevalence of Acute Kidney injury among adults in Ethiopia | 3 |
| **METHODS** | | |  |
| Eligibility criteria | 5 | Those published and/or unpublished original research articles among adult populations in Ethiopia, published in English language, were included in the study. | 6 |
| Information sources | 6 | The procedure for this systematic review and meta-analysis was designed in accordance with the Preferred Reporting Item for Systematic Review and Meta-Analysis (PRISMA) guideline([14](#_ENREF_14))(Liberati, 2009 #1). We searched on PubMed, Google Scholar, Web of Science, and Google for grey literature from articles reporting on acute kidney injury among people age greater than or equal to 18 years. Endnote Version 7 reference management software to download, organize, review, and cite the articles. | 4,5 |
| Search strategy | 7 | The search string is stated as “Acute kidney injury” OR AKI OR “renal impairment” OR “renal dysfunction” OR “renal disease” AND “Human Immuno-deficiency Virus patients” OR “HIV patients” OR “HIV positive patient*” OR “Sero-positive patients” OR “Acquired Immune Deficiency Syndrome patients” OR “AIDS patients” OR “people living with HIV/AIDS” OR PLWHA AND Africa | 4 |
| Selection process | 8 | Articles that reported the /prevalence of acute kidney injury were included. Conference papers, articles without full text, and published in languages other than English were excluded in the study. | 4 |
| Data collection process | 9 | Two reviewers (YMF and HKA) screened the titles, abstracts, and full texts of the included studies. Any controversy was resolved by consensus. An effort was made to communicate with the authors whenever further information was needed. The data were extracted independently by these two reviewers. Using Microsoft excel, data on the author(s), study year/year of publication, study design, sample size, and prevalence of acute kidney injury were extracted. The entire manuscript was critically reviewed and approved by all the authors for publication. | 6 |
| Data items | 10a | Articles reported the prevalence, magnitude, incidence were used for this reviewed study based on the AKIN definition of AKI. | 6 |
|  | 10b | All relevant study characteristics were included | 6 |
| Study risk of bias assessment | 11 | .Begg’s and Egge’sr test was computed to detect publication bias | 7 |
| Effect measures | 12 | The proportion (p), logp, standard error of p, and logodds of outcome measures were used presentation of results. | 7 |
| Synthesis methods | 13a | Those full text articles, and publish in English language were the criteria for the synthesis of this study. | 7 |
|  | 13b | Both the sample size, and the proportion(p) were used for data presentation or evidence synthesis. | 7 |
|  | 13c | PRISMA flow chart, forest plot, and Funnel plot were used to present visually displayed data. | 4 |
|  | 13d | Funnel plot and Q statistics (**I**^2^) was used to assess the heterogeneity status of the included studies. A random effect model was used to treat potential biases. | 7 |
|  | 13e | The heterogeneity is suboptimal the Authors did not use further analysis | 7 |
|  | 13f | Sensitivity analysis was carried out. | 7 |
| Reporting bias assessment | 14 | . Begg’s and Egger’s with p-value <0.005 and or 95% CI were used to declare the presence of publication bias | 7 |
| Certainty assessment | 15 | The pooled summary effect size of the study was used; heterogeneity and possible bias were assessed. | 7 |
| **RESULTS** | | |  |
| Study selection | 16a | The search strategy retrieved 819 research articles. After the removal of duplicated articles, 41 articles remained for further screening. About 9 full-text articles were accessed for eligibility, of which one article was excluded because of reporting without the outcome of interest. Finally, eight studies were retrieved and included with the total sample of 2822 populations. | 7 |
|  | 16b | Studies were excluded because of outcome interest and not full text articles. | 7 |
| Study characteristics | 17 | Finally, twenty-four articles were retrieved and included in the review, with a total of 9713 populations. Of the twenty-four studies, five were from Ethiopia [[19-23](#_ENREF_19)], four from Tanzania [[24-27](#_ENREF_24)], four from Nigeria [[28-31](#_ENREF_28)], two from Uganda [[32](#_ENREF_32), [33](#_ENREF_33)], one from Kenya [[34](#_ENREF_34)], one from Cameroon [[35](#_ENREF_35)], one from South Africa [[36](#_ENREF_36)], one from Malawi [[37](#_ENREF_37)], one from Senegal [[38](#_ENREF_38)], one from Zambia [[39](#_ENREF_39)], one from Rwanda [[40](#_ENREF_40)], one from Burkina Faso [[41](#_ENREF_41)], and one from DR Congo [[42](#_ENREF_42)]. The pooled prevalence was calculated from the aforementioned studies, whereas for predictors of AKI, three studies for hemoglobin level [[19](#_ENREF_19), [35](#_ENREF_35), [41](#_ENREF_41)], six studies for CD4 count [[19](#_ENREF_19), [20](#_ENREF_20), [24-26](#_ENREF_24), [31](#_ENREF_31)], and three studies for WHO clinical HIV staging [[20](#_ENREF_20), [26](#_ENREF_26), [35](#_ENREF_35)] were used. The prevalence of AKI among HIV patients ranged from 2.53% in Uganda [[33](#_ENREF_33)] to 56.8% in Nigeria [[31](#_ENREF_31)]. The majority of the studies used lab tests as a method of data collection. All of the included studies had high-quality scores (**Table 2**). | 7 |
| Risk of bias in studies | 18 | Newcastle Ottawa assessment scale adapted from the cohort and cross-sectional studies quality assessment tools with a score of 5 out of 10 considered a high quality score. | 6 |
| Results of individual studies | 19 | **Table** **2:** Characteristics and quality status of included studies | 8 |
| Results of syntheses | 20a | The risk of bias was assed using Newcastle Ottawa quality assessment tool | 6 |
|  | 20b | The pooled prevalence of acute kidney injury was found to be 19.49% (95% CI: 15.812%-23.169%), I^2^ = 84.8%, and p-value <0.001. | 8 |
|  | 20c | The Heterogeneity of was carried out but it is not suboptimal | 8 |
|  | 20d | The sensitivity analysis indicates there is no study away from the lower and upper confidence intervals. Most of the studies concentrated around the points of the estimation | 9 |
| Reporting biases | 21 | Regarding publication bias, the p-value in the Begg’s test is 0.003, and in Egger’s tests the 95% CI is between 0.31 and 0.62 which is significant and indicates there is a publication bias. | 8 |
| Certainty of evidence | 22 | Considering the publication bias, trim and fill analysis were computed, making a total of ten articles | 8 |
| **DISCUSSION** | | |  |
| Discussion | 23a | This systematic review and meta-analysis revealed that the overall prevalence of acute kidney injury was found to be 19.49%. The pooled prevalence of acute kidney failure was found to be high. Acute kidney injury has been the leading public health concern in recent decades, so efforts should be made to reduce its occurrence, severity, and complications. Early identification and treatment of the underlying causes are prompt actions to tackle the disease progression and burden | 9 |
|  | 23b | The study includes both articles studied on the incidence and prevalence of acute kidney injury, which may lower the overall pooled prevalence of acute kidney injury. | 9 |
|  | 23c | The current study did not include the factors towards | 10 |
|  | 23d | Furthermore, the study was conducted on different population groups in a hospital-based setting irrespective of admission unit | 10 |
| **OTHER INFORMATION** | | |  |
| Registration and protocol | 24a | The review was not still register, it is in progress |  |
|  | 24b | The review protocol has been registered in PROSPERO with reference number; CRD42023446078 |  |
|  | 24c | Further amendments may /not needed. |  |
| Support | 25 | The Authors did not receive any fund for this particular study. |  |
| Competing interests | 26 | There is no competing of interest. |  |
| Availability of data, code and other materials | 27 | The data extracted from included studies and data used for all analyses are submitted as supporting information. |  |

*From:* Page MJ, McKenzie JE, Bossuyt PM, Boutron I, Hoffmann TC, Mulrow CD, et al. The PRISMA 2020 statement: an updated guideline for reporting systematic reviews. BMJ 2021;372:n71.doi: 10.1136/bmj.n71

For more information, visit:<http://www.prisma-statement.org/>
