# Supplementary material for: Acute kidney injury and its predictors among HIV-positive patients in Africa: Systematic review and meta-analysis
Source: PLoS One. 2024 Feb 9;19(2):e0298302. doi: 10.1371/journal.pone.0298302 (PMC10857608; doi:10.1371/journal.pone.0298302)
Supplement: S2 File — (DOCX) [file pone.0298302.s002.docx]

**Supporting information-search strategy**

| **S no** | **Database** | **Search strategy** | **Search result** |
| --- | --- | --- | --- |
| 1 | Pubmed  (by, title, abstract or fulltext | “Acute kidney injury” OR AKI OR “renal impairment*” OR “renal dysfunction*” OR “renal disease” AND “Human Immuno-deficiency virus patient*” OR“HIV patient*” OR “HIV positive patient*”OR “ Sero-positive patient*” OR “Acquired Immune Deficiency Syndrome patient*” OR“AIDS patient*” OR “people living with HIV/AIDS” OR PLWHA AND Africa OR Angola OR South Africa OR Ethiopia OR Egypt OR Morocco OR Sudan OR Senegal OR Nigeria OR DR Congo OR Algeria OR Zambia OR Mozambique OR Sri Lanka OR Cameroon OR Tanzania OR Uganda OR Rwanda OR Burkina Faso OR Malawi OR Tunisia OR Kenya OR Niger OR Sierra Leone OR Eritrea OR Chad OR Cote d'Ivoire OR Djibouti OR Gambia OR Ghana OR Liberia OR Mali OR Togo OR Somalia OR Zimbabwe OR Benin OR Burundi | 253 |
| 2 | Embas | “Acute kidney injury” OR AKI OR “renal adj3 impairment*” OR “renal adj3 dysfunction*” OR “renal adj3 disease” AND “human immune-deficiency virus patient*” OR“HIV patient*” OR “HIV positive patient*”OR “Sero-positive patient*” OR “Acquired immune-Deficiency syndrome patient*” OR“AIDS patient*” OR “people living with HIV/AIDS” OR PLWHA* AND Africa OR Angola OR South Africa OR Ethiopia OR Egypt OR Morocco OR Sudan OR Senegal OR Nigeria OR DR Congo OR Algeria OR Zambia OR Mozambique OR Sri Lanka OR Cameroon OR Tanzania OR Uganda OR Rwanda OR Burkina Faso OR Malawi OR Tunisia OR Kenya OR Niger OR Sierra Leone OR Eritrea OR Chad OR Cote d'Ivoire OR Djibouti OR Gambia OR Ghana OR Liberia OR Mali OR Togo OR Somalia OR Zimbabwe OR Benin OR Burundi | 127 |
| 3 | EBSCO  (title or abstract) | “Acute kidney injury” OR AKI OR “renal N1impairment*” OR “renal N1 dysfunction*” OR “renalN1 disease” AND “human immune-deficiency virus patient*” OR“HIV patient*” OR “HIV positive patient*”OR “Sero N0 positive patient*” OR “Acquired immunodeficiency syndrome patient*” OR“AIDS patient*” OR “people living with HIV/AIDS” OR PLWHA AND Africa OR Angola OR South Africa OR Ethiopia OR Egypt OR Morocco OR Sudan OR Senegal OR Nigeria OR DR Congo OR Algeria OR Zambia OR Mozambique OR Sri Lanka OR Cameroon OR Tanzania OR Uganda OR Rwanda OR Burkina Faso OR Malawi OR Tunisia OR Kenya OR Niger OR Sierra Leone OR Eritrea OR Chad OR Cote d'Ivoire OR Djibouti OR Gambia OR Ghana OR Liberia OR Mali OR Togo OR Somalia OR Zimbabwe OR Benin OR Burundi | 93 |
| 4 | OVID | “Acute kidney injury” OR AKI OR “renal adj3 impairment$” OR “renal adj3 dysfunction$” OR “renal adj3 disease” AND “human immune-deficiency virus patient$” OR“HIV patient$” OR “HIV positive patient$”OR “Sero adj3 positive patient$” OR “Acquired Immune Deficiency Syndrome patient$” OR“AIDS patient$” OR “people living with HIV/AIDS” OR PLWHA AND Africa OR Angola OR South Africa OR Ethiopia OR Egypt OR Morocco OR Sudan OR Senegal OR Nigeria OR DR Congo OR Algeria OR Zambia OR Mozambique OR Sri Lanka OR Cameroon OR Tanzania OR Uganda OR Rwanda OR Burkina Faso OR Malawi OR Tunisia OR Kenya OR Niger OR Sierra Leone OR Eritrea OR Chad OR Cote d'Ivoire OR Djibouti OR Gambia OR Ghana OR Liberia OR Mali OR Togo OR Somalia OR Zimbabwe OR Benin OR Burundi | 76 |
| 5 | Cochrane Library | “Acute kidney injury” OR AKI OR “renal impairment*” OR “renal dysfunction*” OR “renal disease” AND “human immune-deficiency virus patient*” OR“HIV patient*” OR “HIV positive patient*”OR “Sero-positive patient*” OR “Acquired immunodeficiency syndrome patient*” OR“AIDS patient*” OR “people living with HIV/AIDS” OR PLWHA AND Africa OR Angola OR South Africa OR Ethiopia OR Egypt OR Morocco OR Sudan OR Senegal OR Nigeria OR DR Congo OR Algeria OR Zambia OR Mozambique OR Sri Lanka OR Cameroon OR Tanzania OR Uganda OR Rwanda OR Burkina Faso OR Malawi OR Tunisia OR Kenya OR Niger OR Sierra Leone OR Eritrea OR Chad OR Cote d'Ivoire OR Djibouti OR Gambia OR Ghana OR Liberia OR Mali OR Togo OR Somalia OR Zimbabwe OR Benin OR Burundi | 32 |
| 6 | Google Scholar | “Acute kidney injury” OR AKI OR “renal impairment” OR “renal dysfunction” OR “renal disease” AND “ HIV positive patients“ OR people living with HIV/AIDS” OR PLWHA AND Africa OR Angola OR South Africa OR Ethiopia OR Egypt OR Morocco OR Sudan OR Senegal OR Nigeria OR DR Congo OR Algeria OR Zambia OR Mozambique OR Sri Lanka OR Cameroon OR Tanzania OR Uganda OR Rwanda OR Burkina Faso OR Malawi OR Tunisia OR Kenya OR Niger OR Sierra Leone OR Eritrea OR Chad OR Cote d'Ivoire OR Djibouti OR Gambia OR Ghana OR Liberia OR Mali OR Togo OR Somalia OR Zimbabwe OR Benin OR Burundi | 215 |
| 7 | Google | Acute kidney injury and HIV and Africa(including list of African countries as stated above) | 275 |

**Notes**:- Truncation *and $, proximity: Adj3, N0 and N1, Boolean operation: AND and OR
