# Supplementary material for: Acute kidney injury and its predictors among HIV-positive patients in Africa: Systematic review and meta-analysis
Source: PLoS One. 2024 Feb 9;19(2):e0298302. doi: 10.1371/journal.pone.0298302 (PMC10857608; doi:10.1371/journal.pone.0298302)
Supplement: S3 File — (DOCX) [file pone.0298302.s003.docx]

Data availability statements for prevalence of Acute kidney injury among HIV patients

| **Authors** | **Year** | **Country** | **population** | **Study**  **design** | **Data**  **collection** | **Funding** | **Sample**  **size** | **Prevalence**  (%) | **population**  **with AKI** | **standard error of prevalence** | **Quality**  **score** |
| --- | --- | --- | --- | --- | --- | --- | --- | --- | --- | --- | --- |
| Alex MT et al. | 2022 | Cameroon | HIV | Cohort | Lab test, interview | Not reported | 206 | 30.6 | 63 | 3.21075257 | 7 |
| Fiseha T & Gebreweld A. | 2021 | Ethiopia | HIV | Cohort | Record review | Not funded | 353 | 22.1 | 78 | 2.20839885 | 8 |
| Karoney MJ et al. | 2022 | Kenya | HIV | Cross-sectional | Lab test, interview | EDCTP | 261 | 10 | 26 | 1.85695338 | 9 |
| Kefeni BT, et al. | 2021 | Ethiopia | HIV | Cross-sectional | Record review,  lab test | JU | 352 | 20.7 | 73 | 2.15948621 | 7.5 |
| Kimweri D, et al. | 2021 | Uganda | HIV | Cohort | Interview, record review, lab test | not funded | 384 | 19.2 | 74 | 2.00997512 | 8 |
| Mwanja MN et al. | 2022 | Tanzania | HIV | CRoss-sectional | Record review,  lab test | TFEL | 396 | 20.7 | 82 | 2.03598312 | 8.5 |
| Mwemezi O, et al. | 2020 | Tanzania | HIV | Cross-sectional | Record review,  lab test | Not funded | 287 | 32.8 | 94 | 2.77128129 | 9 |
| Mugabo C & Ndikubwimana I. | 2023 | Rwanda | HIV | Cohort | record review | Not reported | 98 | 24.4 | 24 | 4.33853168 | 8.5 |
| Fall K et al. | 2017 | Senegal | HIV | Cohort | Interview, record review, lab test | not reported | 248 | 12.9 | 32 | 2.12852175 | 8 |
| Nyende L et al. | 2020 | Uganda | HIV | cross sectional | Record review | Mulago KCCA project | 278 | 2.53 | 7 | 0.94183179 | 7 |
| Enyew K, et al. | 2016 | Ethiopia | HIV | Cross-sectional | lab test | AAU | 60 | 23.3 | 14 | 5.45757883 | 7.5 |
| Mapesi H, et al. | 2021 | Tanzania | HIV | Cohort | lab test | MOH T | 556 | 7.4 | 41 | 1.11015586 | 7 |
| Vachiat AI, et al. | 2013 | South Africa | HIV | cross-sectional | Record review | Not reported | 101 | 21 | 21 | 4.05286844 | 8 |
| Ali Y, et al. | 2012 | Ethiopia | HIV | Cross-sectional | Interview, record review, lab test | Not reported | 321 | 15.9 | 51 | 2.04100488 | 9 |
| Semde A, et al. | 2023 | Burkina Faso | HIV | Cross-sectional | Record review | Not reported | 364 | 29.94 | 109 | 2.40054733 | 8.5 |
| Emem CP, et al. | 2008 | Nigeria | HIV | cross sectional | lab test | Not reported | 400 | 38 | 152 | 2.42693222 | 7 |
| Maina MS, et al. | 2023 | Nigeria | HIV | Case-control | lab test | Not reported | 200 | 10.5 | 21 | 2.16766003 | 7.5 |
| Struik GM, et al. | 2015 | Malawi | HIV | cross-sectional | lab test | Not reported | 526 | 23.3 | 123 | 1.84324375 | 8 |
| yilma D, et al. | 2012 | Ethiopia | HIV | Cross-sectional | lab test | FAD | 340 | 26.9 | 91 | 2.40489085 | 9 |
| Okafor UH, et al. | 2015 | Nigeria | HIV | Cross-sctional | Interview, record review, lab test | Not reported | 383 | 53.3 | 204 | 2.54931005 | 9 |
| Sakajiki, et al. | 2011 | Nigeria | HIV | Cross-sectional | Interview, record review, lab test | Not reported | 240 | 56.8 | 227 | 3.19749902 | 7.5 |
| Kilonzo BS, et al. | 2016 | Tanzania | HIV | cross-sectional | Interview, record review, lab test | Not reported | 637 | 28 | 179 | 1.77899836 | 8 |
| Tembo S, et al. | 2017 | Zambia | HIV | Cross-sectional | lab test | Not reported | 360 | 24.76 | 89 | 2.27482747 | 9 |
| Ekat MH, et al. | 2012 | DR Congo | HIV | Cross-sectional | lab test | No reported | 562 | 8.5 | 48 | 1.17639143 | 8.5 |

**Notes**:- EDC: European and Developing Countries, HIV: Human immunodeficiency virus; TFEL- Tanzania Field Epidemiology and Laboratory, MOH T- Ministry of Health Tanzania, FAD-Foreign affairs of Denmark

Data availability statement for predictors

1. Hemoglobin level

| Authors | year of publication | Country | Population | study design | Factor | **Odds ratio** | **95% Confidence interval** | |
| --- | --- | --- | --- | --- | --- | --- | --- | --- |
| Alex MT et al | 2022 | Cameroon | HIV | Cohort | Hgb | 3.5 | 1.7 | 7.4 |
| Fiseha T & Gebreweld A | 2021 | Ethiopia | HIV | Cohort | Hgb | 2.19 | 1.16 | 4.09 |
| Semde A, et al | 2023 | Burkina Faso | HIV | Cross-sectional | Hgb | 1.14 | 0.29 | 4.39 |

**Notes**: CI- confidence nterval, HIV-Human immune deficiency virus, WHO- World health organization, OR- Odds ratio

1. CD4 cunt

| Authors | year of publication | Country | Population | study design | Factor | **Odds ratio** | **95% Confidence interval** | |
| --- | --- | --- | --- | --- | --- | --- | --- | --- |
| Fiseha T & Gebreweld A | 2021 | Ethiopia | HIV | Cohort | CD4 count | 2.75 | 1.4 | 5.42 |
| Kefeni BT, et al | 2021 | Ethiopia | HIV | Cross-sectional | CD4 count | 6.47 | 3.31 | 12.64 |
| Kimweri D, et al | 2021 | Uganda | HIV | Cohort | CD4 count | 0.63 | 0.24 | 1.65 |
| Mwemezi O, et al | 2020 | Tanzania | HIV | Cross-sectional | CD4 count | 1.25 | 0.67 | 2.3 |
| Mapesi H, et al | 2021 | Tanzania | HIV | Cohort | CD4 count | 1.01 | 0.54 | 1.9 |
| Sakajiki, et al | 2011 | Nigeria | HIV |  | CD4 count | 3.44 | 2.204 | 5.392 |

**Notes**: CI- confidence nterval, HIV-Human immune deficiency virus, WHO- World health organization, OR- Odds ratio

WHO clinical HIV stages

| Authors | year of publication | Country | Population | study design | Factor | **OR** | **95% CI** | |
| --- | --- | --- | --- | --- | --- | --- | --- | --- |
| Alex MT et al | 2022 | Cameroon | HIV | Cohort | WHO clinical stage of HIV/AIDS | 3.3 | 1.4 | 7.8 |
| Kefeni BT, et al | 2021 | Ethiopia | HIV | Cross-sectional | CD4 count | 2.39 | 1.27 | 4.39 |
| Mapesi H, et al | 2021 | Tanzania | HIV | Cohort | CD4 count | 1.74 | 0.89 | 3. 41 |

**Notes**: CI- confidence interval, HIV-Human immune deficiency virus, WHO- World health organization, OR- Odds ratio
