# Supplementary material for: Acute kidney injury and its predictors among HIV-positive patients in Africa: Systematic review and meta-analysis
Source: PLoS One. 2024 Feb 9;19(2):e0298302. doi: 10.1371/journal.pone.0298302 (PMC10857608; doi:10.1371/journal.pone.0298302)
Supplement: Attachment — Submitted filename: Response to Reviewers.docx [file pone.0298302.s004.docx]

**Response to reviewers**

| S. No | **Comments to the Author** | | **Author Response** | | | |
| --- | --- | --- | --- | --- | --- | --- |
| 1 | Is the manuscript technically sound, and do the data support the conclusions? **Reviewer #1: Partly** | | **Yes, the manuscript is technically sound and the data support the conclusion.** Because, the manuscript aimed to investigate the burden of acute kidney injury and its predictors among HIV positive patients. Based on these objectives the authors investigated the pooled prevalence of AKI and its predictors to resolve the inconsistencies across studies. The authors prepared data extraction format to extract appropriate data from the eligible studies. Finally, the authors used standard procedures/statistical methods to investigate the AKI prevalence and predictors (**as described in the method section**). Considering these, the conclusions are drawn based on the findings of the pooled prevalence and the pooled effect of the predictors. | | | |
| 2 | Has the statistical analysis been performed appropriately and rigorously? **Reviewer #1: I Don't Know** | | **Yes, the statistical analysis has been performed appropriately and rigorously.** The quality of the included studies was assessed using the Newcastle-Ottawa Scale assessment tool. The authors used a random effect model of meta-analysis. The variation/heterogeneity of the studies was investigated using Cochrane Q statistics and I-square test. The biases of the studies were evaluated using the visual inspection of the funnel plot and/or the Egger’s test. To detect the effect of single study, further analysis such as sensitivity analysis were carried out. Furthermore, Trim and fill analysis were conducted to treat the potential publication bias. Lastly, meta-regression analysis was computed to see the effect of predictor variables on AKI. **As a result, the authors performed appropriate statistical analysis.** | | | |
| 3 | Have the authors made all data underlying the findings in their manuscript fully available? **Reviewer #1: No** | | **Yes**, **because the data were provided based on the PLOS data policy:** The data that support the findings are summarized in the manuscript. In addition, the data were provided as part of its supporting information and shared to the public without restrictions | | | |
| 4 | 4. Is the manuscript presented in an intelligible fashion and written in standard English? **Reviewer #1: Yes** | | Thank you very much | | | |
| **Reviewer #1 questions and comments** | | | | | | |
| Clarity regarding for the following points | | | | | | |
| 1 | The PECO/PEO framework | | | The author used PEO framework | |  |
| 2 | Lines 116-119: AKI is defined according to AKIN criteria; however, it is not clear from the extracted/or included articles if all utilized AKIN in their original articles | | | It is edited and corrected. The AKI definition criteria from the previous studies were based on AKIN, KDIGO, and/ or eGFR (**Table 2)** | | |
| 3 | In terms of other confounding factors in the patients included what type of ARTs were they on? Any other medications and/or co morbidities? Who ended up on RRT? | | | The authors tried to investigate ARTs as first line or second line, TDF based or Non TDF based, other nephrotoxic medication use, and presence of comorbidities (Yes/No), but due inconsistent use of those variables by the previous authors we omit those variables to prevent small study bias. | | |
| 4 | Other variables: why was hemoglobin, CD4 count, WHO clinical staging only variables chosen? | | | Different researchers reported the effect of hemoglobin, CD4 count, and WHO clinical staging, but the findings were inconsistent across the studies, as a result the authors investigate the pooled effects those variables on acute kidney injury among HIV patients. | | |
| 5 | consistency with referencing (others in CAPITAL LETTERS) | | | It is edited and corrected ( **Ref. 23, 31, and 41**) | | |
| **Additional editor comments** | | | | | | |
|  | | The authors are requested to review their data search strategy separate from the inclusion and exclusion criteria. It is not clear the characteristics of manuscripts they included in their data search. The authors are also requested to share an extensive search strategy that they used (for any one of the databases they used). | | | The search strategy is attached at file attachment section as **supporting information**. | |
