# Supplementary material for: Acute kidney injury and its predictors among HIV-positive patients in Africa: Systematic review and meta-analysis
Source: PLoS One. 2024 Feb 9;19(2):e0298302. doi: 10.1371/journal.pone.0298302 (PMC10857608; doi:10.1371/journal.pone.0298302)
Supplement: Attachment — Submitted filename: Response to Reviewers.docx [file pone.0298302.s005.docx]

**Response to reviewers**

| S. No | **Comments to the Author** | **Author Response** |
| --- | --- | --- |
| 1 | **Additional Editor Comments:** The paper is a great addition to the literature on acute kidney injury. The authors are applauded on highlighting this disease process in HIV positive patients in Africa. The systematic review was performed in an applaudable manner. The authors are requested to submit the paper to a language editor. The paper is accepted with minor revision as indicated. | Ok, thank you very much. Based on your recommendation the whole manuscript edited using the professional language editor.  **See the entire manuscript please.** |
| 1 | **Comments to the Author** If the authors have adequately addressed your comments raised in a previous round of review and you feel that this manuscript is now acceptable for publication, you may indicate that here to bypass the “Comments to the Author” section, enter your conflict of interest statement in the “Confidential to Editor” section, and submit your "Accept" recommendation.  Reviewer #1: All comments have been addressed | Thank you very much |
| 2 | Is the manuscript technically sound, and do the data support the conclusions?  The manuscript must describe a technically sound piece of scientific research with data that supports the conclusions. Experiments must have been conducted rigorously, with appropriate controls, replication, and sample sizes. The conclusions must be drawn appropriately based on the data presented.  Reviewer #1: Yes | Thank you very much |
| 3 | Has the statistical analysis been performed appropriately and rigorously?  Reviewer #1: Yes | Thank you very much |
| 4 | Have the authors made all data underlying the findings in their manuscript fully available?  Reviewer #1: Yes | Thank you very much |
| 5 | 5. Is the manuscript presented in an intelligible fashion and written in standard English?  Reviewer #1: Yes | Thank you very much |
| 6 | Review Comments to the Author  Reviewer #1: Thank you for a well presented research paper. Hoping that the results of this systematic review will encourage prospective work from the African perspective. | Thank you very much |
